# Collaborations on blood transfusion research in sub‐Saharan Africa: who, what and where

**DOI:** 10.1111/vox.12884

**Published:** 2020-02-05

**Authors:** Amelia Fisher, Selina Wallis, Oliver Hassall, Russell Martin, Imelda Bates

**Affiliations:** ^1^ St James’s University Hospital University of Leeds Leeds UK; ^2^ University of Liverpool Liverpool UK; ^3^ Liverpool School of Tropical Medicine Liverpool UK; ^4^ Department of Computer Science University of Liverpool and The Data Incubator Washington DC

**Keywords:** Sub‐Saharan Africa, blood transfusion, research, collaborations

## Abstract

**Background and objectives:**

Children and pregnant women use 75% of the blood supply in sub‐Saharan Africa (SSA) but face widespread blood shortages. To increase safe blood supply, Africa‐specific evidence and strengthened capacity for transfusion research are needed. Our study analysed seven years of SSA transfusion publications, compared researched topics against priorities and enumerated SSA transfusion research collaborations.

**Materials and methods:**

Data on research topic, journal type, authors’ institutions and country were extracted from transfusion‐related SSA articles published between 2008 and 14 and used to construct a quantitative, graphic visualization of collaborations. Research topics were compared to those identified as priorities for SSA blood services in 2008 and 2015.

**Results:**

Of the 2176, 267 articles (average 38/year) met criteria for analysis. They involved 1245 authors, 673 institutions, 59 countries (35 SSA) and 1375 collaborations. About 41% were on transfusion‐transmitted infections. About 34% were published in specialist transfusion journals. Only 7% involved exclusively collaborations within SSA. Two of the top fifteen institutions by publication quantity were from outside SSA.

**Conclusion:**

Despite a general paucity of SSA‐relevant transfusion research, Francophone SSA was well‐represented. Published research topics are not well matched to SSA research priorities; research on supply, distribution, financing and systems is particularly neglected. The study provides a baseline against which to track any refocusing of research activity to better meet SSA’s needs. Transfusion research hubs within and beyond SSA have been identified as a springboard network for expanding SSA transfusion research capacity.

## Introduction

Blood transfusion services are critical for achieving the Sustainable Development Goals particularly for children and pregnant women with severe anaemia and haemorrhage 1. Sub‐Saharan Africa (SSA)1Sub‐Saharan Africa is defined by the World Bank as 48 out of all 54 countries in Africa (i.e. excluding Algeria, Djibouti, Egypt, Libya, Morocco and Tunisia). https://data.worldbank.org/region/sub-saharan-africa
 still has some of the worst health indicators for these two groups. Children and pregnant women use 75% of SSA’s blood supply, predominantly for emergencies 2, 3. There is a sevenfold difference in blood donation rates between high‐ and low‐income countries (33·1 v 4·6 donations/1000 people) 4. Despite a 25% increase in the number of blood donations globally from 2004 to 2014, widespread blood shortages persist in SSA and contribute to unnecessary illness and deaths 2, 5, 6.

Blood services are expected to provide universal access to safe and effective blood and blood products for transfusion to meet the needs of their national population 4. The infrastructure and management systems needed to achieve this are very complex. Blood services must attract and retain donors and provide quality‐assured testing to maximize blood safety. They have to store the blood safely and in some cases manage the distribution and cold chain to transport the blood and components to hospitals. Every component should be traceable from donation to transfusion or discard. Blood services’ operations are strongly influenced by the resources available. In poorer countries, blood is used predominantly for emergencies, blood services often rely on manual rather than electronic records, they use more whole blood than components, and blood donations and transfusions often occur within a single hospital rather than through a centralized or national blood service.

Globally, the vast majority of evidence used to guide blood services’ practices and policies has been generated by wealthy countries where the organization of blood services and the clinical use of blood are very different from those in poorer countries. SSA‐specific transfusion research is scarce. In 2008, a workshop for blood service leaders, policymakers, blood users and industry was held in Mombasa, Kenya, to agree a list of priority transfusion research topics for SSA 7. Critically, the workshop identified that there was only a handful of individuals in SSA able to carry out this research, and at a subsequent workshop in Pretoria, South Africa, in 2015 to update the transfusion research priorities, the scarcity of transfusion researchers in SSA was again highlighted 8. Although there are very few full‐time researchers employed within blood services in SSA, many of the clinical and laboratory staff, and service managers, are likely to have an interest in research in order to improve their practice. While blood services do have access to research infrastructure such as laboratory and data handing facilities, they may not have access to other types of support required for some aspects of research such as preparing grant applications or financial management of projects. In the last decade, there have been a couple of initiatives to improve research capacity in SSA such as the *Building research capacity of blood transfusion services in Africa programme* (T‐REC), which involved the African Society for Blood Transfusion, and the *Francophone Africa Blood Transfusion Research Network* 8, 9. However, until its own research capacity is substantially expanded, SSA’s transfusion research agenda will remain largely unfulfilled.

An effective way to address the chronic and critical lack of priority‐focused transfusion research capacity within SSA would be to expand existing research collaborations within (i.e. south–south) and beyond, Africa (i.e. south–north). The objectives of this study were to use bibliographic analysis of publications as a novel approach for documenting existing collaborations and networks for blood transfusion research in SSA and to explore researchers’ productivity, the type and frequency of topics that have been researched.

Data were drawn from published articles on blood transfusion topics involving SSA between January 2008 and December 2014. This period covered the seven years between the two workshops. We obtained information from the publications concerning which countries, institutions and researchers in SSA were involved in blood services research and created a quantitative visualization of these research collaborations. The aim was to create a baseline against which subsequent changes in the profile and extent of transfusion research collaborations and networks could be compared. A secondary aim was to identify successful collaborations that could be expanded and modelled elsewhere. In addition, by matching the transfusion research topics covered in the global literature against the stated priorities of SSA’s transfusion community, research topics that were priorities, but which had been neglected, could be identified, and become targets for future research efforts and investments.

## Materials and Methods

A comprehensive search of the Medline, Web of Science and EBSCO Global Health databases using English search terms was conducted to identify published articles on blood transfusion and blood services using a set of consensus search words. This strategy was able to identify papers written in French if they had an English abstract. Articles were selected if they were peer‐reviewed, involved primary research on blood services and/or blood transfusion (excluding plasma exchange and haemodilution), had been conducted in SSA or used transfusion‐related biological or clinical data from human participants in SSA and were published between 1 January 2008 and 31 December 2014. The World Health Organization definition for SSA was used so the literature search covered all African countries south of the Sahara.

### Search strategy

Terms relating to blood transfusion and blood banking (with wildcards when necessary) and individual names for countries in SSA were used to search the electronic databases (Supplementary File S1). A ‘search diary’ was created detailing the names of the databases searched, the keywords used and the search results. Titles and abstracts of studies to be considered for retrieval and reasons for inclusion or exclusion were recorded in an Endnote database. Articles were selected for retrieval after abstracts and titles had been screened for relevance to the study objectives; duplicate articles were removed. Two authors (IB and OH) independently assessed retrieved articles and agreed on those to be included in the study.

### Data extraction and winnowing

Information on authors, authors’ institutions, journal, year of publication, h5‐index, country of study, study design, research topics and key findings was recorded for all articles on a pre‐designed matrix. The h‐index attempts to measure scientific productivity and impact of a researcher and is based on the set of the researcher's most cited papers and the number of citations that they have received in other people's publications. It is an automatically calculated metric available through, for example, Scopus or Google Scholar 10. The h5‐index refers to publications within the 5 years prior to the date of the calculation and was used in preference to the total h‐index to reduce bias due to the variety in the length of careers of the researchers. The research topics of each article were independently identified by at least two authors (from SW, AF, OH and IB) and were initially categorized under the five themes from the 2008 SSA research priorities agenda (i.e. biological safety; blood donors; hospital use of blood; supply and distribution; and systems and financing) 7. Discordances were resolved through discussions among at least three authors (SW, AF, IB and OH), and additional themes were identified so that all research topics from the selected articles could be categorized. Data were winnowed by removing duplicates and by ensuring consistency in British/US spellings and by translating French institutions’ names into English.

### Bibliographic analysis

The institutional affiliation and country location of the authors for each article were extracted from the data set and used to explore the network of collaborations between institutions. A graphic visualization of collaborations was constructed from the data using ‘Python programming language’ and ‘networkx’ package (version 2.2) 11. Python is an interpreted, high‐level, general‐purpose programming language. It produces a graph in which institutes are ‘nodes’, and two institutes are connected if there is one (or more) paper(s) co‐authored by researchers affiliated to those institutions. ‘Self‐loops’, where two co‐authors belong to the same institute, are ignored. The resultant graph visually represents the relative importance of members of a network as brokers of research since an institution with a high number of connections is responsible for a large transfer of ‘items’ (i.e. publications) through the network 12.

## Results

### Research focus of the eligible publications

**Figure 1:**
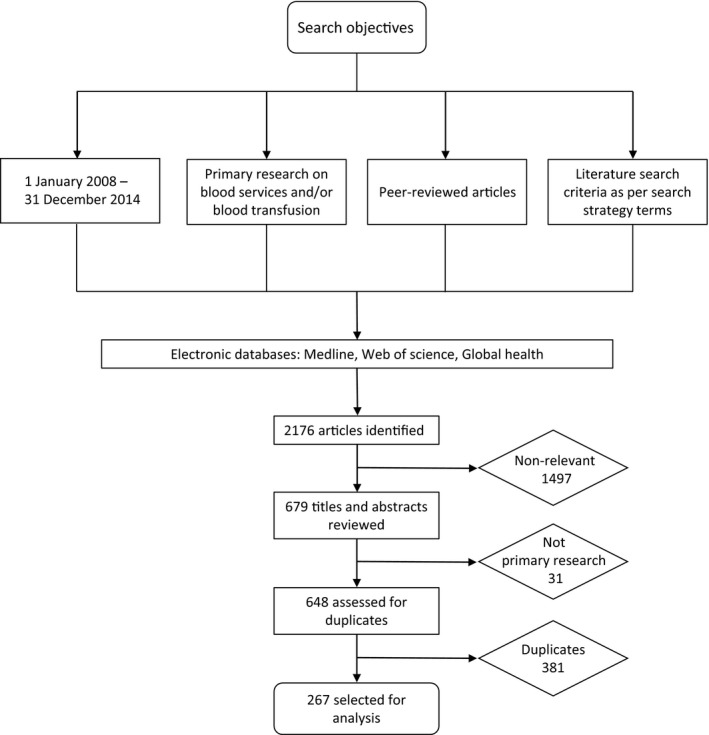
Flow diagram for articles selected for inclusion in the study.

The initial literature search yielded 2176 articles, and after removal of non‐relevant articles, duplicates and non‐primary research, 267 articles were included in the final analysis (Fig. 1; Supplementary File S2). Three new themes – ‘blood groups/immunogenetics’, ‘blood sparing/saving interventions at patient level (e.g. red cell salvage) and at population level’ (e.g. impact of national‐level programmes on transfusion rates) and ‘use of blood donor blood for biomedical research’ – were added to the original 2008 research themes. These were research topics that were identified within the publications but which had not been included in the original 2008 research themes. The original research themes had been based on the experiences and knowledge of the workshop participants rather than a review of the literature. It is possible that these new themes may reflect emerging new technologies. The categorization of themes was subjective and determined through discussion between authors, and it was felt that these three new themes did not fit within other themes and that they would be overlooked if they were not identified separately.

On average, 38 articles had been published on transfusion in SSA each year over the 7 years. There had been little change in the annual number of transfusion publications or research topics since 2008 (Table 1) apart from a transient increase in articles on biological safety in 2009. The ‘biological safety’ theme (theme 1) comprised almost half of all articles (46%; 124/267); to facilitate analysis, this theme was subdivided into four subthemes (Table 1). About 81% of articles under ‘biological safety’ (41% of all included articles) focused on transfusion‐transmitted infections (1a). On average, less than two papers per year were published on each of ‘adequate supplies and equitable distribution’ (theme 4), ‘transfusion systems and sustainable financing’ (theme 5), ‘blood groups/immunogenetics’ (theme 7) and ‘blood sparing’ (theme 8).

**Table 1:** Blood transfusion research themes in publications from SSA, 2008‐2014

Theme	Definition	Total	%
1 Biological safety		124	47
1a TTIs	The prevalence and effects of transfusion‐transmitted infections especially HIV. The effectiveness of screening for blood‐borne viral, bacterial and protozoal infections	109	41
1b Haemovigilance	Monitoring of blood transfusion reactions	6	2
1c Blood grouping	The extent, risks and consequences of blood group incompatibilities	2	1
1d Storage, contamination and red cell abnormalities in donated blood	The extent, risks and consequences of bacteriological infections, blood storage methods, refrigeration and cold chains	7	3
2 Blood donors	Family, replacement and voluntary donors. Incentives, disincentives and motivations for donation. Blood donor health. Culturally appropriate education messages and strategies to increase donations	23	9
3 Hospital management of blood and blood transfusion	Guidelines for the clinical use of blood, effect of implementing, training methods for safety, barriers responsible for delays in the management of blood stocks and issuing and transportation of blood to the wards	34	13
4 Adequate supplies and equitable distribution	Creating targets for blood collection in Africa to plan and budget accurately for transfusion services. Distance to transfusion centre equity. Calculating necessary number of units collected for clinical outcomes and protecting donors	9	3
5 Transfusion systems and sustainable financing	Health economics and decision analysis to inform decision makers of the costs and consequences blood transfusion interventions including improving quality systems for blood group screening and infection transmission. Evaluation of and transferability of the various service delivery models in the African context. Poverty and unplanned costs of emergency blood transfusion. Sustainability of National transfusion systems financed by external donors. Barriers to sustainability and alternative macro‐and micro financing methods. Private health providers access to and involvement with blood transfusion services	11	4
6 Use of blood donor blood for biomedical research	Blood donor samples used for population‐based prevalence, incidence or genomic studies, or development of reference ranges	43	15
7 Blood groups/immunogenetics	Alloimmunization and red cell phenotypes	10	4
8 Blood sparing (at individual or programme level)	Use of ancillary treatments, such as fluid replacement, and optimizing the ratios and rates of fluid and blood infused to lower overall blood use. The most effective therapeutic packages for those in need of emergency transfusions have not been determined in resource‐poor settings	13	5
Total		267	100

### Countries in SSA where authors’ institutions were located

The authors’ institutions were located in 35 countries in SSA (Table 2); eleven were francophone, and one was lusophone, and the rest were anglophone. In addition, there were seven francophone and six anglophone multicountry articles. West Africa (totalling 18 countries)2
https://www.worldatlas.com/articles/the-regions-of-africa.html
 produced the most publications (165, 62%), followed by east Africa (totalling 14 countries) (39, 15%), southern Africa (totalling 10 countries) (30, 11%) and central Africa (totalling 6 countries) (26, 10%). Thirty per cent (79/267) of all articles were from Nigeria, and five countries (Nigeria, Ghana, South Africa, Uganda and Cameroon) accounted for 58% (156/267) of the single country articles. The median h5‐index of authors from five countries was 50 or above. These were as follows: Namibia (median h5 62; 3 articles), Zambia (51; 1 article), South Africa (50; 16 articles), Mozambique (50; 5 articles) and Cote d’Ivoire (50; 3 articles). Of the top five countries ranked by number of articles, South African researchers had the highest median h5‐index (50).

**Table 2:** Countries in SSA in which research on blood services and transfusion was conducted from 2008 to 2014 and journal authors’ h5‐index

Country	Language (if not English)	Number (%) of papers	Authors’ median h5‐index
Nigeria		79 (30)	12
Ghana		35 (13)	36
South Africa		16 (6)	50
Uganda		14 (5)	27·5
Cameroon	French	12 (4)	17
Burkina Faso		11 (4)	24
Congo	French	11 (4)	12
Kenya		11 (4)	34
Mali	French	11 (4)	12
Malawi		6 (2)	17
Tanzania		6 (2)	23·5
Mozambique	Portuguese	5 (2)	50
Benin	French	5 (2)	12
Ethiopia		4 (1)	13
Togo	French	4 (1)	14·5
Cote d'Ivoire	French	3 (1)	50
Namibia		3 (1)	62
Senegal	French	3 (1)	12
Zimbabwe		3 (1)	38
Gabon	French	2 (0·7)	18
Guinea	French	2 (0·7)	49
Mauritania	French	2 (0·7)	33
Niger	French	2 (0·7)	30
Botswana		1 (0·4)	23
Madagascar		1 (0·4)	12
Rwanda		1 (0·4)	11
Zambia		1 (0·4)	51
Francophone SSA^a^	French	7 (3)	50
Anglophone SSA^a^		6 (2)	50
Total		267	

a Multicentre studies.

### Journals that have published research on blood services and transfusion in SSA 2008‐2014

Articles were published in both specialist transfusion journals (91/267, 34%) and non‐transfusion journals (176/267, 66%) (Table 3). Five specialist transfusion journals and six non‐transfusion journals each published at least five of the articles (a total of 60 and 39 articles, respectively). Of these journals that published five or more articles, 38% of articles (35/91) were published in Transfusion (the journal of the American Association of Blood Banks; h5‐index 50) and 13% (12/91) were published in Vox Sanguinis (journal of International Society of Blood Transfusion; h5‐index 34). Transfusion was the specialist journal that published the most articles (35), followed by Transfusion Clinique et Biologique (17). A total of 137 articles were published in non‐transfusion journals, and each of these journals published less than five articles on blood services and transfusion.

**Table 3:** Journals that published five or more articles on blood services or blood transfusion from SSA between 2008 and 2014 (note: 137 non‐transfusion journals each published less than 5 transfusion articles: data not shown in table)

Journal	No. papers	Authors’ h5‐index
Specialist transfusion Journals		
Transfusion	35	50
Transfusion Clinique et Biologique	17	12
Transfusion Medicine	14	17
Vox Sanguinis	12	34
Blood Transfusion	9	24
Subtotal of specialist transfusion journals that published at least 5 articles	87	
Transfusion and Apheresis Science	3	21
Asian Journal of Transfusion Science	1	12
Subtotal	91	
Non‐transfusion journals		
Nigerian Journal of Clinical Practice	10	12
PLoS ONE	8	148
Pan African Medical Journal	6	12
Malaria Journal	5	51
Nigerian Journal of Medicine: Journal of the National Association of Resident Doctors of Nigeria	5	11
Nigerian Postgraduate Medical Journal	5	7
Subtotal of non‐transfusion journals that published at least 5 articles	39	

Non‐transfusion journals publishing 5 or more papers accounted for 39 papers (15%). The h5‐index of the authors in these journals ranged from 2 to 148. A total of 239 authors in these publications had h5‐indices between 0 and 50, and 26 had H5‐indices between 51 and 150. The h5‐index was not found for 2/267 articles. The top three journals by authors’ h5‐index were all non‐transfusion journals – PLOS One (h5 148; 8 papers, Hepatology (127; 1 paper) and Clinical Infectious Diseases (113; 1 paper).

### Institutional affiliations of authors of articles on blood transfusion in SSA 2008‐2014

Forty per cent (106/267) of articles were published jointly from institutions in Africa and in the global north (i.e. south–north collaboration) and 7% (18/267) were collaborations between institutions exclusively in different African countries (i.e. south–south collaboration). When ranked by the number of publications, two of the top fifteen institutions were from outside SSA (Table 4). Komfo Anokye Teaching Hospital, Ghana, ranked highest among the African institutions with 18 articles: six (33%) had a first author from SSA, and 15 (83%) involved a south–north collaboration. The University of Cambridge, UK, also had 18 articles, and 17 of these had at least one co‐author from an African institution. Five of the top fifteen institutions by number of articles, all from Nigeria, did not have any external institutional collaborations.

**Table 4:** Frequency of institutional affiliations of first and last authors of articles on blood transfusion in SSA 2008‐2014

Institution	Total number of publications	Number of publications in which the first author was from this institution	Number of publications in which the last author was from this institution	Number of publications in which first and last authors were from this institution	Number of south–north institutional collaborations in these publications	Number of south–south institutional collaborations in these publications	Median authors’ H5‐index
Univ Cambridge, Cambridge, UK	18	12	10	7	17	0	50
Komfo Anokye Teaching Hospital, Kumasi, Ghana	18	6	5	1	15	0	50
Niger Delta Univ, Wilberforce Isl, Bayelsa State, Nigeria	9	8	5	5	0	0	22
Centers for Disease Control and Prevention, Atlanta, GA, USA	9	6	8	6	8	2	47
Ctr Natl Transfus Sanguine, Bamako, Mali	9	5	2	2	6	2	12
National Blood Transfusion Center, Ouagadougou, Burkina Faso	9	4	0	0	4	5	43
Makerere Univ, Kampala, Uganda	8	7	1	7	7	0	28
Obafemi Awolowo Univ, Ife, Osun State, Nigeria	8	7	6	6	0	0	14
Kenya Govt Med Res Ctr, Ctr Geog Med Res Coast, Kilifi, Kenya	7	4	2	2	6	2	50
South African National Blood Service, Johannesburg, South Africa	7	4	2	1	7	0	50
Ctr Rech Biomol Pietro Annigoni CERBA, Ouagadougou, Burkina Faso.	6	4	6	4	1	3	18
Univ Benin, Benin, Nigeria	6	4	5	4	0	0	9
University of Ghana, Accra, Ghana	6	4	3	3	2	0	43
Lagos State Univ, Teaching Hosp, Ikeja, Lagos State, Nigeria	5	4	0	0	0	0	11
University of Nigeria Enugu Campus, Enugu, Nigeria	5	4	4	4	0	0	11

### Visualization of the network of publication collaborations

**Figure 2:**
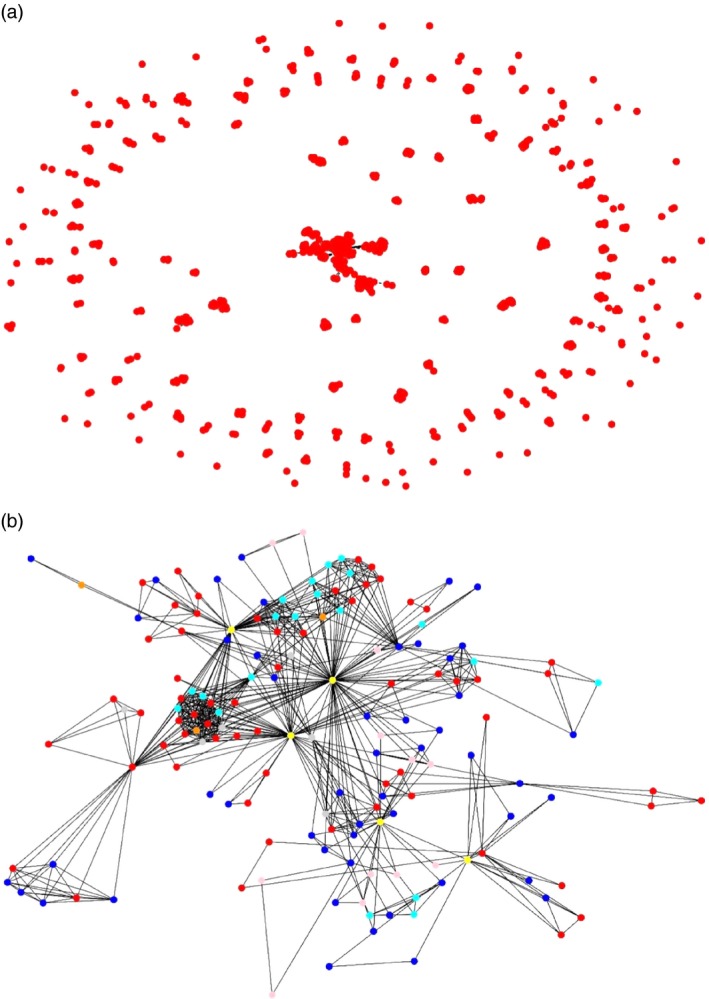
Graphic of collaborations in transfusion publications 1 January 2008‐31 December 2014. (a) This graphic has 671 nodes (each represented by a red circle), which are institutions with unique names, and 1375 ‘virtual’ (i.e. invisible) lines. Each virtual line represents a collaboration between two institution where there is at least one paper co‐authored by researchers from the corresponding institutions. (b) This is an exploded view of the 165 institutions in the centre of Fig. 2a. Institutions with only one collaborative transfusion publication and ‘self‐loops’ which involve only authors from the same institution have been excluded leaving 163 institutions represented in the figure. The five institutions with the most ‘connectedness’ (i.e. the largest betweenness centrality) are shown in yellow. Blood Systems Research Institute (BSRI), San Francisco, USA, has the most ‘connectedness, and the other four yellow nodes are Centre National de Transfusion Sanguine, Bamako, Mali; Centre National Transfusion Sanguine, Ouagadougou, Burkina Faso; Univ Calif San Francisco, San Francisco, CA, USA and Centre National Transfusion Sanguine, Nouakchott, Mauritania. Other institutes in Africa are in red and non‐African institutes are in blue. Key to colours of nodes by African region (for categorization of countries, see Supplementary File S5). Yellow, top_five_nodes in terms of connectedness (i.e. betweenness centrality); Pink, southern_Africa; Cyan, central_Africa; Dark orange, eastern_Africa; Red, western_Africa; Silver, northern_Africa; Blue, non_African_countries.

The data from the 267 articles used to construct the graph of the network collaborations (Fig. 2a and b) were based on 1245 authors, 673 institutions and 59 countries (35 African and 24 non‐African). There are 1375 lines indicating interinstitutional collaborations involving 67 sets of connected institutions and 50 unconnected institutions (i.e. isolated nodes) (Fig. 2a). To provide more detail about the range and intensity of collaborations, collaborations that only occurred once (i.e. ‘node with degree 1’) were removed so that each remaining institution had at least two collaborations (i.e. ‘node with degree ≥2’); this resulted in inclusion of 163 institutions (Fig. 2b). The top five institutions with the most collaborations were in the USA (2), Mali, South Africa and the UK with normalized ‘connectedness’ values varying between 1·00 (ranked first) and 0·47 (ranked fifth) (Supplementary Files S3 and S4).

## Discussion

### Mismatch between SSA research priorities and published topics

This study identified 267 articles on blood transfusion and blood services in SSA published during the 7‐year period. Almost half of the articles focused on just one of the eight themes (biological safety), and of these, over 80% focused on transfusion‐transmitted infections. Very little had been published on other research topics identified by SSA transfusion stakeholders as priorities, such as blood sparing, supply and distribution of blood, and systems and financing 8. This bias in research focus towards transfusion‐transmitted infections during the study period (2008‐2014) is not surprising as it coincides with the HIV epidemic in Africa. It may be related to asymmetries in influence in financial resources, technical expertise and indirect financial and political incentives between external donors and domestic stakeholders 13. The PEPFAR initiative focused on 14 countries with a high HIV burden, 12 in Africa, and aimed to provide development assistance rather than research funds 14, 15. PEPFAR was responsible for the majority of the USD 0·6‐2·1 billion allocated to global blood safety programs, particularly HIV screening, between 2000 and 2015. The timescale of our study was contemporaneous with the 2010‐2015 funding peak, and our data therefore provide a novel platform from which to compare changes in transfusion research activity and focus as PEPFAR funding is withdrawn from these countries. In particular, it will be critical to evaluate changes in the quantity of publications and the portfolio of research topics covered in the period since 2015 to document any refocusing towards the other, non‐HIV, research priorities already identified by the African transfusion community.

### Countries, journals and research partnerships

Articles on transfusion in SSA were distributed between specialist and non‐specialist transfusion journals. By country, South African authors had both the highest number of articles and h5‐index ≥50. The h5‐index varies between different research disciplines and grows as citations accumulate so it depends on the ‘academic age’ of a researcher. The higher values of h5‐index found in our study (e.g. Table 2) may therefore partially reflect that some countries have a more long‐standing and larger pool of transfusion researchers who have been publishing for many years. A large proportion of articles were from francophone countries possibly related to the existence of a specialist Francophone transfusion journal (Transfusion Clinique et Biologique) and the fact that the AfSBT produces outputs in French and English and alternates its meetings between anglophone and francophone countries. This language equity is encouraging since the francophone linguistic community is often under‐represented in global health but comprises over one‐third of UN member states and half the world’s countries with the lowest Human Development Index 16.

Our study has uncovered examples of long‐standing and successful collaborations between academic and health services institutions. Such academic–service partnerships are important for blood transfusion research in SSA due to the scarcity of national funding available for research and the very limited capacity for research within blood services in SSA 17. The blood services have a wealth of well‐documented data and access to a ‘normal’ population through their blood donors which should make them attractive research partners 17. Ensuring that such partnerships are equitable is vital to generate excellent science and obtain the best development outcomes where all partners benefit from the research. A substantial proportion of articles in our study involved south–north collaborations but only 7% involved institutional collaborations exclusively between African institutions. This is similar to previous findings from a study in Southern Africa where only 3%–5% of articles involved intra‐Africa collaborations, and there was an ‘unbalanced and unequal partnership‘ between South Africa and other countries in the region 18. Blood services in SSA highlighted the need for collaboration at both the 2008 and 2015 transfusion research workshops so that together they could generate evidence to overcome their common challenges. International collaborations are important for improving visibility and recognition, utilizing expensive equipment and facilities, and acquiring expertise and new ideas for research. However, it is important to ensure that such collaborations do not divert ‘southern’ partners’ resources away from national priorities. It is also likely that some of the successful collaborations we identified were initially driven by one or two highly productive researchers, and it will be interesting to track how these collaborations between individuals and institutions develop in the future. The presence of motivated and academically qualified staff in the blood services and a conducive environment in which they can be productive are also important for enhancing the research capacity of blood services in SSA.

Studies on how to promote equitable collaborations between developed and developing country researchers have identified factors which balance the imperatives of research with capacity strengthening and strategic development priorities 19, 20. Specifically, the eight factors underpinning sustainable, effective and equitable global health research collaborations are – involvement in cutting‐edge, interesting science; effective leadership; competence and commitment to good scientific practice; capacity building; respect for the needs and interests of partners; opportunities for discussion and disagreement; trust and confidence; and justice and fairness 21. Power differentials in south–north research partnerships are reducing as scientists in developed countries have increasingly recognized the influence of local context on their work, and developing countries have built up their own research capabilities. South–south research collaborations have also grown as developing countries have sought to strengthen their science base 20. Funders and professional organizations also have an essential role to play throughout the research lifecycle to select and build partnerships of equals to foster equity 22.

To strengthen transfusion research capacity in SSA, it is important to identify existing successful research leaders and to support them to establish hubs for training other researchers and for generating evidence to inform African transfusion policies and practice. Our study has identified transfusion researchers who are based in African institutions and has linked this information to evidence of the quality of their publications in terms of h5‐index. Traditionally, research performance has been evaluated using citation count, quality and quantity of research output but these metrics do not take any account of the role of collaborations which are critical in ensuring high quality and sustainable research. We used quantitative social network mapping approach to measure ‘collaboration supportiveness’ and to enhance understanding about how the transfusion research collaborations in SSA operate, and which institutions and researchers are the most active and well connected 23, 24. This new information about the quality and quantity of transfusion publications produced by individual researchers and their institutions will be useful for research funders and researchers looking for new partnerships or networks for transfusion research in SSA. It also provides evidence for researchers and institutions about the importance of their contribution to transfusion research outputs.

### Strengths and limitations

We conducted a rigorous literature search based on PRISMA guidelines and pre‐defined criteria to identify articles to include in the study 25. We used English (British and American) search terms so it is possible we may have missed some non‐anglophone articles. Articles were independently assessed by two authors before being selected for inclusion, and the research focus of the articles was also assessed independently, with a pre‐defined procedure for resolving discrepancies. Nevertheless, a few articles may have been missed or a small number of research topics misclassified. It was not possible to ascertain the nationality of the authors from the articles, so the data can only provide an indication of the research publication activity of African transfusion researchers. There may be some inaccuracies in the number of institutes and authors in the data set due to slight variations in the names of institutions or authors as cited in publications, and some authors may affiliate with more than one institution. Where possible, we minimized these inaccuracies by contacting the authors or by interrogating institutional websites when we suspected that different names may have been used. We also ensured consistency by normalizing American and British spellings, and French and English names for institutions, but some undetected errors may remain.

### Next steps

It is clear from these data that during this seven‐year study period, there was an overriding research focus on transfusion‐transmitted infections, while many other research areas also identified as important by African stakeholders had been overlooked. It would also be interesting to explore the reasons behind the imbalance in research activities and to understand more about the infrastructure, academic environment and collaboration arrangements that are conducive to fostering productive transfusion research in SSA. A workshop in 2017 in the USA identified research opportunities to improve the availability of safe blood and blood components and transfusion practices in Africa and elsewhere where the need for an ‘adequate supply of safe blood’ and the lack of country‐level data was highlighted 26. A mixed approach of needs assessment, implementation research and targeted interventions underpinned by a robust evidence base was recommended to address this need. Research to meet SSA’s needs should be based on the principles of equitable partnerships and accompanied by adequate funds to train, grow, mentor and network a community of African transfusion researchers. Funding for such research will need to come from a mix of national and international sources and would be compatible with the increased recognition that implementation research is critical for bridging the gap between research generation and better health outcomes 27.

Blood services are an essential, life‐saving component of health systems. Our study has shown that currently research activity is poorly matched to SSA’s needs, and there is a significant lack of transfusion research capacity in SSA. This will undoubtedly hinder blood services’ ability to generate evidence which could improve the effectiveness of their operations and improve the supply of safe blood. There have been some recent initiatives to increase the number of transfusion researchers in Africa and to provide them with networking opportunities. These include a programme organized by the International Society of Blood Transfusion (ISBT) which provides training in research skills with much material delivered remotely through webinars to facilitate access, combined with small group interactions and mentorship 28. The African Society for Blood Transfusion has developed a research policy, research and ethics committee and, in collaboration with ISBT, is creating an online research forum and researcher network 29. Our study could be used to develop a ‘road map’ and as a baseline against which to compare any increase and refocussing of transfusion research in SSA over the next few years. These positive initiatives will need significant investment and expansion if they are to bring about the step change in research capacity in SSA that is needed to respond to the challenges of providing adequate supplies of safe blood for the continent.

## Conflict of interest

There are no conflicts identified.

## Funding

European Commission Grant FP7/98388. The funders did not have any role in the writing of the manuscript or the decision to submit it for publication.

## Supporting information

Supplementary File S1 Terms used for literature search.
Supplementary File S2 Database of articles used in literature review.
Supplementary File S3 The top 25 most connected institutions–the highest value indicates the most ‘connectedness’ (i.e. normalized betweenness centralities).
Supplementary File S4 List of the top 26 institutions with the broadest range of different collaborations in transfusion articles published 2008–14, ranked by the number of collaborations.
Supplementary File S5 Coding used to classify institutions according to their African regional location.
