# Supplementary material for: Collaborations on blood transfusion research in sub‐Saharan Africa: who, what and where
Source: Vox Sang. 2020 Feb 5;115(3):221–32. doi: 10.1111/vox.12884 (PMC7187137; doi:10.1111/vox.12884)
Supplement: Supplementary file 1 — Supplementary File S1 Terms used for literature search. Supplementary File S2 Database of articles used in literature review. Supplementary File S3 The top 25 most connected institutions–the highest value indicates the most ‘connectedness’ (i.e. normalized betweenness centralities). Supplementary File S4 List of the top 26 institutions with the broadest range of different collaborations in transfusion articles published 2008–14, ranked by the number of collaborations. Supplementary File S5 Coding used to classify institutions according to their African regional location. [file VOX-115-221-s001.docx]

### Supplementary files

#### Supplementary file 1. Terms used for literature search

| Blood Transfusion and Africa in MEDLINE | |
| --- | --- |
| MeSH terms | Keywords |
| Blood transfusion  (EXPLODE above to include terms below) | “blood transfusion” “transfusion*blood” |
| - Blood component transfusion - (EXPLODE above to include terms below) | “blood component transfusion” “transfusion*blood component” “component transfusion*blood” |
| - - Erythrocyte transfusion   - Leukocyte transfusion   - Platelet transfusion | “transfusion*red blood cell” “transfusion*erythrocyte” “red blood cell transfusion” “erythrocyte transfusion”  “leukocyte transfusion”  “platelet transfusion” “transfusion*platelet” “transfusion*blood platelet” “platelet transfusion*blood” “blood platelet transfusion” |
| - Exchange transfusion, whole blood | “exchange transfusion whole blood” |
| Blood banks | “blood bank” “blood*bank” |
| Blood preservation  (EXPLODE above to include terms below) | “blood preservation” “preservation*blood” |
| - Blood safety | “blood safety” “hemovigilance” “safety blood” |
| Blood donors | “blood donation” “blood donor” “donor*blood” “donation*blood” |
| Transfusion medicine | “medicine transfusion” “transfusion medicine” |
| Blood grouping and crossmatching | “crossmatching blood” “blood crossmatching” “blood typing” “blood grouping” “blood grouping and crossmatching” |
| Blood group incompatibility | “reaction*transfusion” “transfusion reaction” “group incompatibilities blood” “incompatibilities blood group” “blood group incompatibility” “group incompatibility blood” |
| Sub Saharan Africa |  |
| - Africa Central   (EXPLODE above to include terms below)   - - Cameroon   - Central African Republic   - Chad   - Congo   - Democratic Republic of the Congo   - Equatorial Guinea   - Gabon |  |
| - Africa Eastern   (EXPLODE above to include terms below)   - - Burundi   - Djibouti   - Eritrea   - Ethiopia   - Kenya   - Rwanda   - Somalia   - Sudan   - Tanzania   - Uganda |  |
| - Africa Southern   (EXPLODE above to include terms below)   - - Angola   - Botswana   - Lesotho   - Malawi   - Mozambique   - Namibia   - South Africa   - Swaziland   - Zambia   - Zimbabwe |  |
| - Africa western   (EXPLODE above to include terms below)   - - Benin   - Burkina Faso   - Cape Verde   - Cote d’Ivoire   - Gambia   - Ghana   - Guinea   - Guinea-Bissau   - Liberia   - Mali   - Niger   - Nigeria   - Senegal   - Sierra Leone   - Togo |  |

#### Supplementary file 2. Database of articles used in literature review

#### Supplementary file 3. The top 25 most connected institutions – the highest value indicates the most ‘connectedness’ (i.e. normalised betweenness centralities)

|  | **Institution** | **Connectedness (i.e. ‘betweenness centrality’) value** |
| --- | --- | --- |
| 1 | Blood Systems Research, Institute (BSRI), Univ California, San Francisco, CA USA | 1 |
| 2 | Centre National Transfusion Sanguine, Bamako, Mali | 0.6 |
| 3 | South African National Blood Service, Johannesburg, South Africa | 0.5 |
| 4 | Univ Calif San Francisco, San Francisco, CA 94143, USA | 0.48 |
| 5 | Univ Cambridge, Div Transfusion Med, Dept Haematol, Cambridge, UK | 0.47 |
| 6 | Centre National Transfusion Sanguine, Ouagadougou, Burkina Faso | 0.3 |
| 7 | Centre National Transfusion Sanguine, Nouakchott, Mauritania | 0.25 |
| 8 | Univ Calif San Francisco, Dept Obstet Gynecol & Reprod Sci, San Francisco, CA 94143, USA | 0.15 |
| 9 | Inst National Transfusion Sanguine, Paris, France | 0.14 |
| 10 | Department of Obstetrics and Gynecology, Faculty of Medicine, Assiut University Women's Health Center, Assiut, Egypt | 0.08 |
| 11 | Komfo Anokye Teaching Hospital, Kumasi, Ghana | 0.07 |
| 12 | Lelie Research, Paris, France | 0.07 |
| 13 | Odontostomatology, Bamako, Mali, and University of Alabama at Birmingham USA | 0.07 |
| 14 | Katsina General Hospital, Katsina, Nigeria | 0.05 |
| 15 | Division of Transfusion Medicine, Department of Haematology, University of Cambridge, Cambridge, UK | 0.05 |
| 16 | South African National Blood Service, Roodepoort, South Africa | 0.04 |
| 17 | South African National Blood Service, Weltevreden Park, South Africa | 0.03 |
| 18 | Transfusion Medicine Unit, Komfo Anokye Teaching Hospital, Kumasi, Ghana | 0.02 |
| 19 | Centre National Transfusion Sanguine, Brazzaville, Democratic Republic of Congo | 0.02 |
| 20 | Agence Nationale pour la Transfusion Sanguine (Ministere de la Sante), Cotonou, Benin | 0.01 |
| 21 | Centre National Transfusion Sanguine, Kigali, Rwanda | 0.01 |
| 22 | Department of Microbiology, University of Ghana Medical School, Accra, Ghana | 0.004 |
| 23 | Liverpool Sch Trop Med, Liverpool, UK | 0.003 |
| 24 | Kenya National Blood Transfusion Serv, Nairobi, Kenya | 0.003 |
| 25 | Komfo Anokye Teaching Hospital, Transfusion Med Unit, Kumasi, Ghana | 0.002 |

#### Supplementary file 4. List of the top 26 institutions with the broadest range of different collaborations in transfusion articles published 2008-14, ranked by the number of collaborations

| **Institution** | **Number of different collaborating institutions** |
| --- | --- |
| Blood Systems Research Institute (BSRI), Univ California, San Francisco, CA USA | 67 |
| Centre National Transfusion Sanguine, Bamako, Mali | 55 |
| Centre National Transfusion Sanguine, Ouagadougou, Burkina Faso | 50 |
| Univ Calif San Francisco, San Francisco, CA 94143, USA | 36 |
| Centre National Transfusion Sanguine, Nouakchott, Mauritania | 29 |
| Inst National Transfusion Sanguine, Paris, France | 26 |
| Centre National Transfusion Sanguine, Brazzaville, Democratic Republic of Congo | 24 |
| Centre National Transfusion Sanguine, Kigali, Rwanda | 19 |
| Univ Cambridge, Div Transfusion Med, Dept Haematol, Cambridge, UK | 18 |
| Service Departemental de la Transfusion Sanguine Atlantique Littoral, Cotonou, Benin | 18 |
| Hopital Laquintinie, Douala, Cameroon Hopital General, Yaounde, Cameroon | 18 |
| Hopital Central, Yaounde, Cameroon | 18 |
| Haematology and Blood Bank service, University Hospital Centre, Yaounde, Cameroon | 18 |
| Centre de Sante de Reference, Kidal, Mali | 18 |
| Centre Regional de Transfusion Sanguine, Sfax, Tunisia | 18 |
| Centre Regional de Transfusion Sanguine, Rabat, Morocco | 18 |
| Centre Regional de Transfusion Sanguine, Niamey, Niger | 18 |
| Centre Regional de Transfusion Sanguine, Fada N'Gourma, Burkina Faso | 18 |
| Centre Regional de Transfusion Sanguine, Bobo-Dioulasso, Burkina Faso | 18 |
| Centre National Transfusion Sanguine, Lome, Togo | 18 |
| Centre National Transfusion Sanguine, Kinshasa, Democratic Republic of Congo | 18 |
| Centre National Transfusion Sanguine, Abidjan, Cote d'Ivoire | 18 |
| Centre Hospitalier d'El Maarouf, Comoros | 18 |
| Univ Calif San Francisco, Dept Obstet Gynecol & Reprod Sci, San Francisco, CA 94143, USA | 17 |
| Department of Obstetrics and Gynecology, Faculty of Medicine, Assiut University Women's Health Center, Assiut, Egypt | 14 |
| Agence Nationale pour la Transfusion Sanguine (Ministere de la Sante), Cotonou, Benin | 14 |

#### Supplementary file 5. Coding used to classify institutions according to their African regional location

southern_Africa = [ "Angola", "Botswana", "Lesotho", "Malawi", "Mozambique",  "Namibia", "South Africa", "Swaziland", "Zambia", "Zimbabwe" ]

central_Africa = [ "Central African Republic", "Cameroon", "Congo", "Democratic Republic of Congo", "Equatorial Guinea", "Gabon", "Zaire" ]

eastern_Africa = [ "Burundi", "Djibouti", "Eritrea", "Ethiopia", "Kenya", "Mauritius", "Rwanda", "Somalia", "Sudan", "Tanzania", "Uganda", "Seychelles", "Comoros", "Mayotte", "Réunion", "Madagascar" ]

western_Africa = [ "Burkina Faso", "Benin", "Gambia", "Ghana", "Guinea", "Guinea-Bissau", "Liberia", "Ivory Coast", "Cote d'Ivoire", "Mali", "Mauritania", "Niger", "Nigeria", "Senegal", "Sierra Leone", "Togo", "Cape Verde", "Sāo Tomé and Principe", "Saint Helena" ]

northern_Africa = [ "Egypt", "Tunisia", "Morocco", ]

Note that not all the countries in the sets above were represented in the collection of papers included in the analysis.
